# HIV-1 Fusion Is Blocked through Binding of GB Virus C E2D Peptides to the HIV-1 gp41 Disulfide Loop

**DOI:** 10.1371/journal.pone.0054452

**Published:** 2013-01-22

**Authors:** Kristin Eissmann, Sebastian Mueller, Heinrich Sticht, Susan Jung, Peng Zou, Shibo Jiang, Andrea Gross, Jutta Eichler, Bernhard Fleckenstein, Heide Reil

**Affiliations:** 1 Institute of Virology, Friedrich-Alexander-Universität Erlangen-Nürnberg, Erlangen, Germany; 2 Bioinformatics, Institute of Biochemistry, Friedrich-Alexander-Universität Erlangen-Nürnberg, Erlangen, Germany; 3 Lindsley F. Kimball Research Institute, New York Blood Center, New York, New York, United States of America; 4 Key Laboratory of Medical Molecular Virology of Ministries of Education and Health, Shanghai Medical College, Institute of Medical Microbiology, Fudan University, Shanghai, China; 5 Medicinal Chemistry, Friedrich-Alexander-Universität Erlangen-Nürnberg, Erlangen, Germany; Boston College, United States of America

## Abstract

A strategy for antiviral drug discovery is the elucidation and imitation of viral interference mechanisms. HIV-1 patients benefit from a coinfection with GB Virus C (GBV-C), since HIV-positive individuals with long-term GBV-C viraemia show better survival rates than HIV-1 patients without persisting GBV-C. A direct influence of GBV-C on HIV-1 replication has been shown in coinfection experiments. GBV-C is a human non-pathogenic member of the *flaviviridae* family that can replicate in T and B cells. Therefore, GBV-C shares partly the same ecological niche with HIV-1. In earlier work we have demonstrated that recombinant glycoprotein E2 of GBV-C and peptides derived from the E2 N-terminus interfere with HIV entry. In this study we investigated the underlying mechanism. Performing a virus-cell fusion assay and temperature-arrested HIV-infection kinetics, we provide evidence that the HIV-inhibitory E2 peptides interfere with late HIV-1 entry steps after the engagement of gp120 with CD4 receptor and coreceptor. Binding and competition experiments revealed that the N-terminal E2 peptides bind to the disulfide loop region of HIV-1 transmembrane protein gp41. In conjunction with computational analyses, we identified sequence similarities between the N-termini of GBV-C E2 and the HIV-1 glycoprotein gp120. This similarity appears to enable the GBV-C E2 N-terminus to interact with the HIV-1 gp41 disulfide loop, a crucial domain involved in the gp120-gp41 interface. Furthermore, the results of the present study provide initial proof of concept that peptides targeted to the gp41 disulfide loop are able to inhibit HIV fusion and should inspire the development of this new class of HIV-1 entry inhibitors.

## Introduction

GB virus C (GBV-C) is a common human virus that can be transmitted sexually, parenterally and vertically from mother to child [1], [2]. Infection of immunocompetent individuals usually leads to clearance of GBV-C viraemia within the first years; however, GBV-C can cause persistent infection in approximately 25% of cases [3]. As a member of the *flaviviridae* family, for which a fourth genus, termed *Pegivirus*, was recently proposed, it is related to the hepatitis C virus (HCV) [4]. However, acute and persistent infection with GBV-C does not appear to be associated with hepatitis or any other diseases in humans (reviewed in [5], [6]). The GBV-C genome is a positive-sense, single-stranded RNA (9.4 kb) that contains one long open reading frame (ORF) [7]–[9]. The polyprotein is post-translationally processed by cellular and viral proteases into the structural proteins such as E1 and E2, as well as the nonstructural proteins NS2, NS3, NS4A/B and NS5A/B [10]. In most industrialized countries 1% to 5% of healthy individuals are viraemic for GBV-C and 10% to 15% have developed anti-E2 antibodies as evidence of past GBV-C infection [11]. In contrast to HCV, in the majority of GBV-C infected individuals, the occurrence of anti-E2 antibodies is associated with clearance of the virus [12]. However, GBV-C elimination has been documented without the appearance of anti-E2 antibodies, indicating that a diagnosis based merely on the detection of anti-E2 antibodies in serum might lead to an underestimation of prevalence of prior infection. Based on the common transmission routes, the prevalence of GBV-C in HCV and HIV–1-positive individuals is much higher than in the general population. GBV-C RNA prevalence rates range from 20% to 30% in HCV coinfected individuals and from 15% to 40% in HIV-1 patients (reviewed in [3]). Several, but not all, epidemiological studies and a meta-analysis have reported that GBV-C viraemia has a beneficial effect on the course of HIV-1 disease progression and survival [13]–[20]. Furthermore, mother-to-child transmission of GBV-C reduces the vertical transmission of HIV-1 from GBV-C/HIV-1 coinfected mothers [21], and recently it was reported that accidental GBV-C acquisition via transfusion is associated with a significant reduction in mortality in HIV-infected individuals [22]. GBV-C is a lymphotropic virus that replicates primarily in B and T cells [23]. Therefore, it occupies, at least in part, the same ecological niche as HIV-1. Several effects have been proposed to explain the interference with HIV-1, including induction of chemokines, CD4 receptor and coreceptor modulation, prevention of Th2 cytokine profile, reduction of T cell activation, as well as downmodulation of FAS-mediated apoptosis in GBV-C coinfected individuals [24]–[31]. Cell culture experiments revealed a direct influence on HIV-1 replication by at least two GBV-C proteins, i.e., the nonstructural protein NS5A and the E2 envelope protein [31], [32]. Whereas NS5A leads to decreased HIV-1 receptor expression and stabilization of the Th1 profile, the E2 protein prevents HIV-1 entry by a mechanism as yet unknown [31], [33]–[35]. Using synthetic peptides presenting different regions of E2, we recently demonstrated that this interference with HIV-1 entry can be ascribed to the N-terminal part of the GBV-C E2 protein ranging from residue 29 to 72 (according to GenBank accession no. AF121950) [33]. Two overlapping 20 mer peptides (P4-7 and P6-2) presenting amino acids 37 to 56 (WDRGNVTLLCDCPNGPWVWV) and 45 to 64 (LCDCPNGPWVWVPAFCQAVG), respectively, of the GBV-C E2 protein, were shown to be most potent with IC_50_s between 2 and 0.2 µM in a TZM-bl-based HIV-1 replication assay.

Cell entry of HIV-1 is mediated by noncovalently associated trimers of gp120 and gp41 subunits assembled into functional spikes on the surface of virions. The noncovalent association between the two subunits appears to be maintained by interaction of the N- and C-termini of gp120 with the disulfide loop region of gp41 and parts of the flanking N- and C-terminal heptad repeat regions (NHR and CHR) (reviewed in [36]). The primary receptor for gp120 is CD4, which is expressed on the surface of monocytes, macrophages, and on subsets of dendritic and T cells [37]. Upon engagement of CD4, gp120 undergoes a conformational shift, enabling binding to the chemokine coreceptors CXCR4 or CCR5, respectively [38], [39]. This interaction is thought to induce additional conformational changes in the envelope trimer that result in exposure of the hydrophobic fusion peptide of gp41 and its insertion into the host cell membrane. Subsequently, the NHR and CHR regions of gp41 fold back and interact with each other, in order to form a six-helix bundle (6-HB), that promotes the convergence of the viral and cellular membrane and the formation of the membrane pore (reviewed in [40]).

In this study, we aimed to elucidate the mechanism of action by which GBV-C E2 20 mer peptides P4-7 and 6-2, representing the E2 residues from 37 to 64, inhibit HIV-1 entry. We demonstrate that E2 peptides act late after CD4 and coreceptor engagement via binding the gp41 disulfide loop region, a new promising target for HIV-1 entry inhibition.

## Materials and Methods

### Peptides

N-acetylated or fluorescein-conjugated peptides derived from the GBV-C glycoprotein E2 (P4-7, P4-7s, P6-2, P6-2s, P9, and P28) were obtained from EMC Microcollections (Tübingen, Germany). All other peptides (listed in Table S1) were synthesized by a standard solid-phase FMOC method using a peptide synthesizer. The N- and C-termini of N36 and C34, as well as the gp120 N-terminus peptides, were acetylated and amidated, respectively. The gp41 disulfide peptides (Loop36ox and Loop36s) were N-terminally biotinylated for site-selective attachment to streptavidin-coated assay plates. Loop36o× was cyclized by formation of a disulfide bridge between the two cysteine residues through air oxidation at pH 8. The peptides were purified to homogeneity (>95% purity) by high-performance liquid chromatography and verified by laser desorption mass spectrometry (PerSeptive Biosystems, Framingham, MA) and by ESI mass spectrometry, respectively. Peptide stocks were dissolved in 75% DMSO/H_2_O and diluted for experiments in respective buffers or medium.

### Virus Stocks

HIV-1 virions containing the BlaM-Vpr chimera were produced as previously described [33]. Briefly, 293T cells were co-transfected with pNL4-3 proviral DNA, pCMV-BlaM-Vpr, and pAdVAntage vectors. After 2 days of cultivation, the virus-containing supernatant was centrifuged for 10 min at 300×g to remove cellular debris. The HIV-1 virion-containing supernatant was overlaid onto a 20% sucrose cushion and ultracentrifuged at 35000×g at 4°C for 90 min. The resulting pellet was resuspended in medium and aliquots were frozen at −80°C until usage.

### HIV-1 Receptor Expression

PHA/IL-2-stimulated PBMC or TZM-bl cells were incubated with E2 peptides (25 µM), phorbol 12-myristate 13-acetate (PMA; 40 ng/ml), SDF-1α (1 µg/ml) or RANTES (50 nM) for 6 hr. CD4, CXCR4 and CCR5 surface expression was analyzed by using standard FACS staining protocols. Cells were washed with PBS and incubated for 30 min at 4°C with PE-labeled antibodies (CD4 clone SK3, CXCR4 clone 12G5, and CCR5 clone 2D7, BD Biosciences, Germany). Subsequently, washed cells were resuspended in PBS and analyzed with a FACSCalibur™ flow cytometer (BD Biosciences, Germany).

### HIV-1 Virion-based Fusion Assay

The virion-based fusion assay was performed essentially as described elsewhere [41]. Briefly, 5×10^4^ TZM-bl cells were infected with sucrose-purified HIV-1 NL4-3 virions containing BlaM-Vpr (5 ng p24-Gag per approach). Under temperature-arrested state (TAS) conditions, binding of the HI virions to target cells was allowed at low temperature via spinning inoculation, for which virions were added to cells and centrifuged at 2095×g, 4°C for 30 min. To remove unbound virus particles, cells were washed with cold medium and incubated with 20 µM of each E2 peptide or respective controls (AMD-3100 [10 µM], 2F5 [0.5 µg/ml], b12 [0.3 µg/ml], and B4 [0.3 µg/ml]) at 23°C for 1 h. Virus-cell fusion was initiated by a temperature shift to 37°C for a total of 2 hr. Under standard conditions, E2 peptides, or defined control agents, were added to cell culture shortly before HIV-1 inoculation, and cells were incubated at 37°C for 2 hr. Subsequently, cells were washed with HBSS, loaded with the CCF2-AM substrate, as described by the manufacturer (Invitrogen, Germany), and incubated overnight at 20°C. BlaM activity was quantified using a fluorescence plate reader. The extent of virus-cell fusion was determined from the ratio of blue (460 nm) and green (510 nm) emission upon exciting the cells at 405 nm.

### HIV-1 gp120 Binding to CD4, CCR5, and CXCR4

HIV-1 gp120 binding studies using flow cytometry were performed as previously described [42]. Briefly, supernatants containing soluble Fc-gp120_JRCSF_ fusion protein or the Fc control protein were obtained from transiently transfected 293T cells. For binding studies, 293T cells were transiently transfected with pCD4 expression plasmid or an empty plasmid as control, and preincubated with 40 µM of the E2 peptides, 1 µM sCD4, 3 µg/ml VRC01, or 10 µM of the CCR5 inhibitor TAK-779. Similar amounts of soluble Fc-gp120 or Fc control, were added for 45 min on ice, and cells were washed and stained with anti-human IgG, FITC-conjugated secondary antibody (Dako, Germany) for 30 min at 4°C. Subsequently, washed cells were resuspended in PBS and analyzed with a FACSCalibur™ flow cytometer. Data were collected with BD CellQuest™ and analyzed with FCS Express V3. In parallel, CD4 and CCR5 expression was determined by staining with the antibody clones SK3 and 2D7, respectively (BD Biosciences, Germany).

HIV-1 gp120 binding studies by immunoblotting were performed as previously described [43]. Briefly, 293T cells were transiently transfected with either pCD4 or pCCR5 expression plasmids. Verification of expression was performed 2 days after transfection via flow cytometry. After 2 days of cultivation, the cells were resuspended in binding buffer (50 mM HEPES, 5 mM MgCl_2_, 1 mM CaCl_2_, 5% BSA, 0.1 mM NaN_3_, pH 7.5). In a 50 µl binding assay, either 40 µM TAK-779 or Maraviroc, 0.8 µM sCD4, 40 µg/ml VRC01, 160 µg/ml 447-52D, 200 µg/ml F425B4e8 (all: NIH AIDS Research and Reference Reagent Program), 40 µg/ml 5F3 (Polymun Scientific), 0.6 µM E2_340_-Fc or Fc control, 0.2 mM P4-7, P6-2 or P28 and additionally 10 µg/ml HIV-1_BaL_ or HIV-1_IIIB_ gp120 protein (NIH AIDS Research and Reference Reagent Program) were added. After incubation for 1 h at 37°C, cells were washed with cold PBS and lysed in Triton X-100 and Tween-20 containing buffer. Lysates were separated by a 5% SDS-PAGE and verified with monoclonal gp120 antibody F425 B4a1 (NIH AIDS Research and Reference Reagent Program) or with sheep α-HIV-1-gp120 (Aalto Bio Reagents Ltd.) in Western Blot analyses. HIV-1 gp120 (5 ng) was used as a standard. Detection was achieved with HRP-conjugated anti-human antibody and chemiluminescence reagents.

### HIV-1 gp160 Binding

293T cells were transiently transfected with an HIV-1 Env expression vector, coding for the CXCR4-tropic NL4-3 gp160 precursor (X4*env*) for surface expression of gp120-gp41 Env trimers or empty plasmid as control. After 2 days of cultivation, cells were incubated with 1 µg sCD4 and 1 µM FITC-conjugated E2 peptides for 1 h at 37°C respectively. Subsequently, cells were washed, resuspended in PBS, and analyzed by flow cytometry using a BD™ LSR II flow cytometer (Becton Dickinson Biosciences, Germany). Flow cytometer data were collected with BD FACSDiva™ and analyzed with FCS Express V3. In parallel, gp160 expression was determined by staining with HIV-1 gp120 mAbs (2G12 and 17b) and an anti-human IgG-FITC pAb (Dako, Germany).

### ELISA Procedures

Flat-bottom, 96-well Microtest ELISA plates (Sarstedt, Germany) were coated overnight at 4°C with recombinant HIV-1 gp120 (0.5 µg/ml), gp41 (0.22 µg/ml) proteins, or with streptavidin (4 µg/ml) in coating buffer (0.1 M NaHCO_3_, 0.1 M Na_2_CO_3_, pH 9.87). Nonspecific binding was blocked (blocking buffer: 1% BSA, 0.1% Tween-20 in phosphate buffer, pH 7.2) for 1 h at room temperature, followed by washing steps (wash buffer: 0.1% Tween in phosphate buffer, pH 7.2). Streptavidin-coated plates were then incubated with gp41 disulfide loop peptides (Loop36ox or Loop36s; 2.5 µM). All plates were then incubated with recombinant E2_340_-Fc or Fc protein in serial dilutions in sample buffer (phosphate buffer, pH 7.2) for 3 hr at room temperature. To perform the competitive ELISAs, the recombinant E2_340_-Fc protein was adjusted at 5 µg/ml and the monoclonal gp41 antibodies (246-D, F240, T32, D50, 2F5, 4E10, Chessie 8 [all: NIH AIDS Research and Reference Reagent Program], and 5F3 [Polymun, Austria]) at 100 µM. After washing procedure, the plates were incubated for 1 h with polyclonal rabbit anti-human IgG peroxidase antibody (Dako, Germany) 1∶6000 in sample buffer and washed. Plates were developed with TMB peroxidase substrate solution (KPL, USA), or with OPD/H_2_O_2_ solution in the dark. Absorbances (ODs) were read at 450 or 492 nm, respectively, using a multi-channel photometer (ELISA-Reader AxSYM, Abbott Laboratory, USA).

### Inhibition of 6-HB Formation

Inhibitory activity of the peptides (P4-7, P6-2, and P28) on 6-HB formation was measured by a modified ELISA-based method as previously described [44]. Briefly, 96-well polystyrene plates were coated with the 6-HB-specific monoclonal antibody, NC-1 IgG, (described by [45]) (2 µg/ml in 0.1 M Tris, pH 8.8), and then blocked with 2% non-fat milk. N36 (0.25 µM), the tested peptides and C34 as control were added at graded concentrations into the wells. After incubation for 30 min at 37°C, C34-biotin (0.25 µM) was added, followed by incubation for 45 min at 37°C and washing with wash buffer (PBS containing 0.05% Tween-20) six times. Streptavidin-labeled horseradish peroxidase (Invitrogen, Grand Island, NY) and the substrate 3,3′,5,5′-tetramethylbenzidine (Sigma, St. Louis, MO) were added sequentially. Absorbance at 450 nm (OD_450_) was measured. The percent inhibition of 6-HB formation by the peptides was calculated as described before [46].

### Computational Methods

Globular and disordered regions in E2 were identified using GlobPlot2.3 [47] with standard settings. Secondary structure prediction was performed using the consensus prediction method provided by the NPS@ server [48]. Structural homologs were identified with the bioinfo.pl meta prediction server [49], and modelling was performed with Modeller6.2 [50], using the crystal structure of a single-variable-domain antibody (PDB code: 2Z8W) as template [51]. Sequence comparison between different E2 proteins and between E2 and gp160 was performed with LALIGN (http://www.ch.embnet.org/software/LALIGN_form.html) using the local alignment option. The gp160 signal peptide comprising residues 1 to 30 was removed prior to the similarity searches because it is not present in the mature protein.

### Statistics

All statistical analyses were performed using Wilcoxon rank-sum test (two-tailed).

## Results

### GBV-C E2-derived Peptides do not Alter Cell Surface Presentation of HIV-1 Receptors

**Figure 1:**
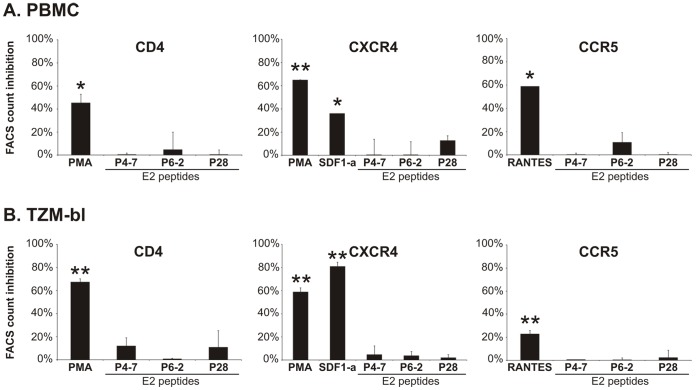
E2-derived peptides do not alter CD4 or coreceptor surface expression. CD4, CXCR4 and CCR5 expression on PBMC (A) and TZM-bl cells (B) after preincubation with E2 peptides were determined by flow cytometry analysis. Positive controls like PMA (phorbol 12-myristate 13-acetate) lead to down-regulation of CD4, SDF-1α (CXCR4-ligand) and RANTES (CCR5-ligand) to down-regulation of the respective coreceptors, while E2 peptides P4-7 and P6-2 including the negative control P28 had no relevant effect on HIV-1 receptor presentation. Percentages illustrate the ratio-to-cell-negative (RTCN) values that were calculated from the percentage of positive cells and the fluorescence intensity of peptide-incubated cells compared to mock-incubated cells. Columns show average values ±SD of two independent experiments each performed in triplicate. *: p<0.05; **: *p*<0.01 (mock- vs. inhibitor-incubated cells).

It has been shown that the HIV-1 receptor/coreceptor density plays a critical role in the efficiency of HIV-1 fusion and infection [52]. Thus, we addressed the question of whether or not the two overlapping GBV-C E2 20 mer peptides P4-7 and P6-2 have an impact on the cell surface presentation of the HIV-1 receptors CD4 and CXCR4 or CCR5, respectively. The cell surface presentation of these receptors was quantified via flow cytometry after incubation of primary peripheral blood mononuclear cells (PBMCs) and of HeLa-derived HIV indicator cells expressing CD4, CCR5 and CXCR4, designated as TZM-bl cells, with the E2 peptides P4-7 and P6-2. The non-HIV-1-inhibitory peptide P28 derived from the C-terminal part of E2 (residue 271 to 290), served as a negative control [33]. As shown in Figure 1, both CD4 and coreceptor expression on PBMC or TZM-bl cells remained largely unaffected by E2 peptides, thereby eliminating a change in receptor density as a mode of action of the E2 peptides.

### The Effect of GBV-C E2 Peptides Arises After gp120/CD4 and gp120/coreceptor engagement

**Figure 2:**
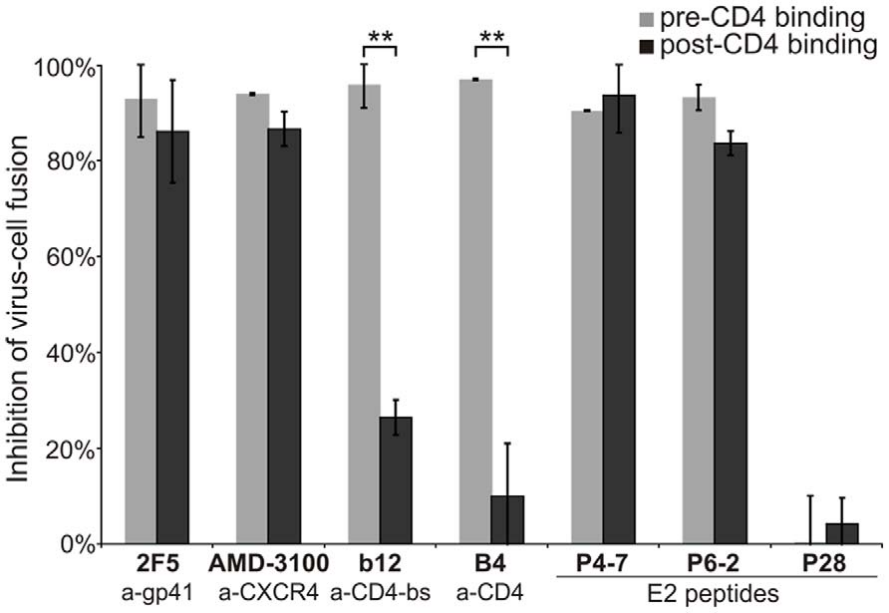
E2-derived peptides inhibit the HIV-1 fusion process. The HIV-inhibitory activity of known HIV-1 entry inhibitors or respective E2 peptides was determined using a virus-cell fusion assay under standard (respective inhibitors were added simultaneously to HIV NL4-3_BlaM-Vpr_ inoculum: pre-CD4 binding) or under TAS (temperature-arrested state) conditions (respective inhibitors were added at low temperature of 23°C for 1 h after spin-inoculation [4°C] and removal of unbound HIV-1 particles: post-CD4 binding). Subsequently, HIV-1 fusion was enabled via a temperature shift to 37°C. Antibodies b12 (anti [a]-CD4-binding site [bs]) and B4 (a-CD4) are early inhibitors interfering with CD4 engagement, whereas 2F5 (MPER antibody) and AMD-3100 (CXCR4 antagonist) act after CD4 binding. The E2 peptide P28 served as a negative control. The extent of virus-cell fusion was determined from the ratio of blue (460 nm) and green (510 nm) emission upon exciting the cells at 405 nm using a fluorescence plate reader. Percentages were calculated in relation to mock-treated cells. Columns show average values ±SD of five independent experiments each performed in triplicate. **: p<0.01.

To find out whether early or late steps of HIV-1 entry are affected by the E2 peptides, the E2 peptide activity was determined before and after gp120/CD4 engagement using the Vpr-β-lactamase (Vpr-BlaM) enzyme-based virus-cell fusion assay under standard (pre-CD4 binding) and temperature-arrested state (TAS; post-CD4 binding) conditions, respectively. Based on the temperature sensitivity of envelope-mediated membrane fusion, low temperature (<28°C) during and after CD4/gp120 engagement leads to an arrest of the fusion process at an intermediate state and the blockade of coreceptor binding, 6-HB formation, and final membrane fusion [53]–[55]. As shown in Figure 2, the effect of antibodies that interfere with the initial CD4/gp120 interaction (b12 and B4) was largely abrogated under TAS conditions. In contrast, P4-7 and P6-2 showed full activity under both conditions, comparable to known fusion inhibitors like AMD-3100 and the neutralizing MPER antibody 2F5 that act after CD4 engagement. Therefore, these data provide evidence that P4-7 and P6-2 implement their HIV-1 inhibitory activity post-CD4 binding of gp120.

**Figure 3:**
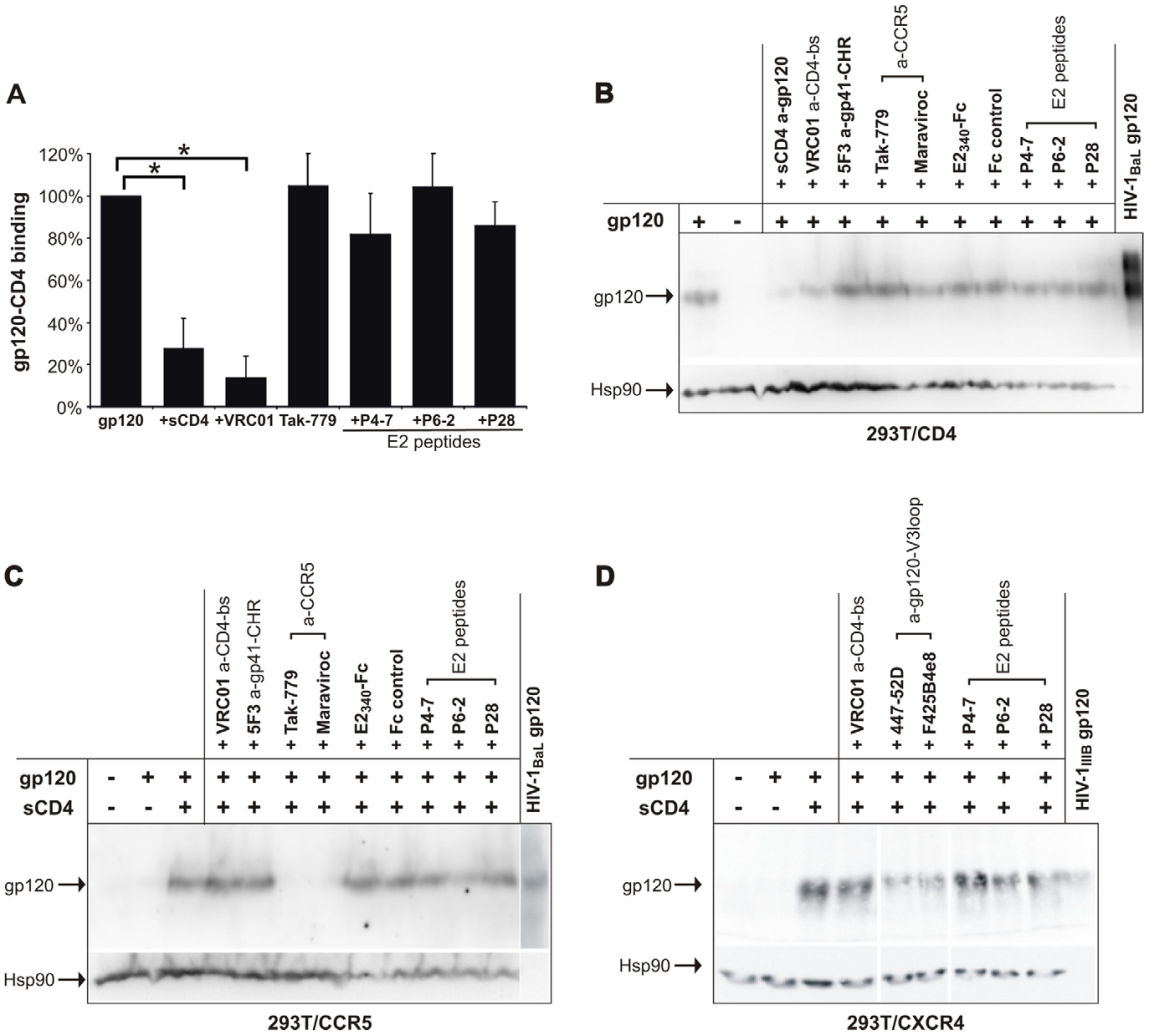
E2-derived peptides do not influence HIV-1 gp120 binding to CD4 and CCR5. (A) 293T cells expressing CD4 were preincubated with E2-derived peptides and specific controls that bind to gp120 (sCD4; VRC01, directed against CD4 binding site [bs]) or CCR5 (Tak-779). Subsequently, cells were incubated with soluble Fc-gp120 (HIV-1_JRCSF_) and binding to CD4 was analyzed by flow cytometry using a FITC-conjugated anti-human IgG pAb. Binding to cells in the absence of inhibitor was set as 100%. Columns show average values ±SD of three independent experiments each performed in duplicate. *: p<0.05 In (B) and (C) 293T cells expressing CD4, CCR5, or CXCR4 were preincubated with E2-derived peptides, E2_340_-Fc and Fc protein as well as specific controls that bind to gp120 (sCD4, VRC01, 447-52D, F425 B4e8), gp41 (5F3), or CCR5 (Tak-779, Maraviroc). Soluble HIV-1 gp120 was added to the CD4-, CCR5-, or CXCR4-expressing cells and binding was investigated by separating the cell lysates on SDS-PAGE and visualized by performing Western Blot analyses. No influence of E2 peptides or intact E2 protein on the binding efficiency between gp120 and CD4, CCR5, or CXCR4 respectively, was observed after normalization of band intensity with the gel loading control Hsp90.

To substantiate this conclusion, next we assessed the impact of E2 peptides on gp120 binding to CD4. Immunoblotting and flow cytometry data revealed that E2 peptides and the whole E2 ectodomain fused to the Fc domain of human IgG (E2_340_-Fc) that showed HIV-1 inhibitory activity in earlier studies [32] do not interfere with the interaction of recombinant gp120 with cellular expressed CD4 (Figure 3A, B). In a related approach, the effect of E2 peptides or intact E2 protein on the interaction between gp120 and the coreceptors CCR5 and CXCR4 expressed on cells was tested by immunoblotting. To ensure efficient binding of gp120 to the coreceptors, soluble CD4 (sCD4) was added simultaneously. Use of CCR5-antagonists Maraviroc and TAK-779 or anti-gp120 coreceptor binding site (V3-loop) antibodies 447-52D or F425B4e8, respectively, as positive controls revealed a clear reduction in the gp120/CCR5 and a less pronounced decline (densitometric analysis ∼50%) in the gp120/CXCR4 interaction. However, after normalization of the band intensity (Hsp90; data not shown) there was no indication for any E2-effect on the binding efficiency between gp120 and the HIV-1 coreceptors CCR5 or CXCR4, respectively (Figure 3C, D).

### Recombinant GBV-C E2 Protein Interacts with gp41, while N-terminal GBV-C E2 Peptides Abrogate this Interaction

**Figure 4:**
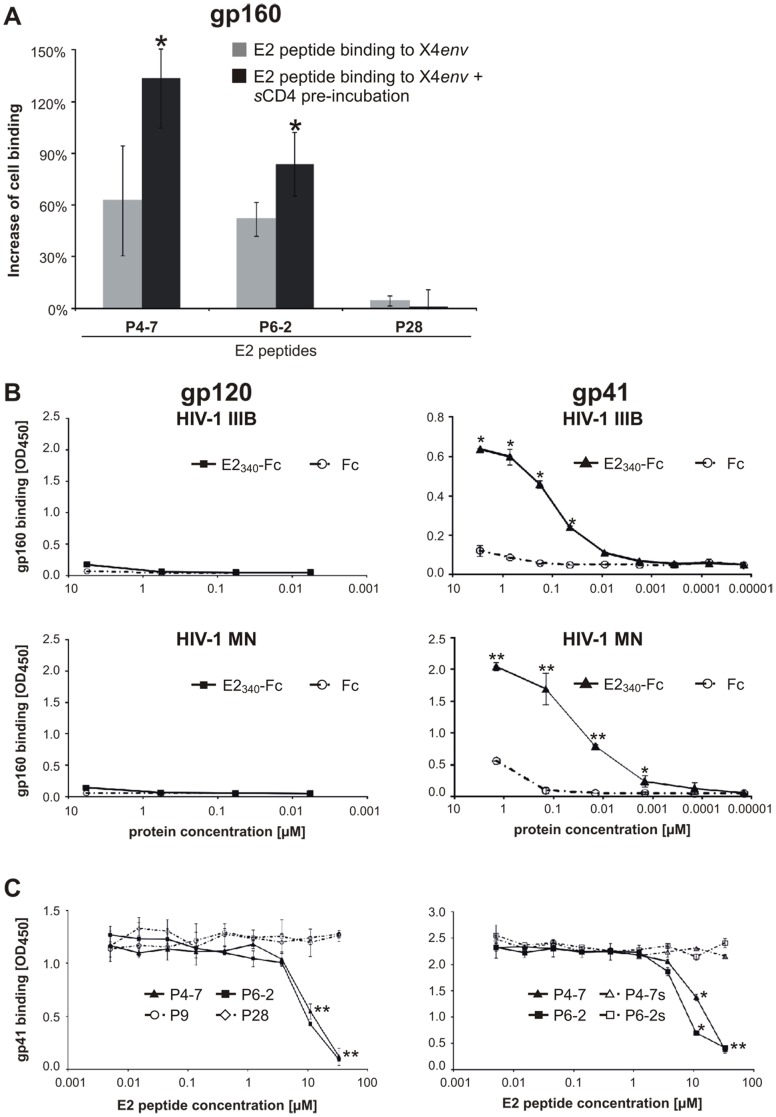
Recombinant E2_340_-Fc protein and E2-derived peptides bind HIV-1 gp41. (A) 293T cells expressing NL4-3 envelope (X4*env*) or empty plasmid as control were incubated with sCD4 and FITC-conjugated E2 peptides, respectively. Peptide binding was analyzed by flow cytometry. Percentages illustrate the increase of cell binding to X4*env*-transfected cells. The increase is normalized to the background binding of E2 peptides to mock-transfected cells by calculating the RTCN value. Columns show average values ±SD of three independent experiments each performed in duplicate. *p<0.05 (vector- vs. X4*env*-transfected cells) (B) Binding of recombinant E2_340_-Fc protein and Fc protein as control to recombinant HIV-1 glycoproteins (gp120_IIIB_, gp120_MN_, gp41_IIIB_, or gp41_MN_). (C) E2 peptides with increasing amounts were added to recombinant E2_340_-Fc protein (at constant concentration) and then transferred to immobilized recombinant gp41_MN_. All ELISA graphs show average values of three independent experiments each performed in duplicate. *: *p*<0.05; **: *p*<0.01 (E2_340_-Fc, Fc, P4-7, or P6-2 vs. P28).

Previously, we have shown that the target of the HIV-1-inhibitory E2 peptides is most likely located on the HIV-1 particle, rather than on the cell [33]. The surface of the HIV-1 particle consists of a lipid bilayer with incorporated host cell proteins and a limited number of functional envelope (Env) trimers (on average 14±7) [56]. Therefore, we tested whether the HIV-1-inhibitory E2 peptides interact with trimeric HIV-1 Env proteins expressed on the surface of transfected cells. For this purpose, 293T cells were transiently transfected with an HIV-1 Env expression vector, coding for the gp160 precursor of the CXCR4-tropic NL4-3 envelope (X4*env*). Binding of FITC-labeled E2 peptides was monitored by flow cytometry. Although we could observe a background attachment of P4-7 and P6-2 to mock- or vector-transfected 293T cells, there was a significant increase in the binding of P4-7 and P6-2 when cells expressed HIV-1 Env and were pretreated with sCD4 (Figure 4A). In contrast, the increase of binding was not observed with the negative control peptide P28. These data suggest that the HIV-inhibitory E2 peptides bind to HIV-1 Env and that this interaction seems to be CD4-dependent, since the induction of conformational changes in gp120 and/or the increase in availability of gp41 after CD4 engagement facilitates E2-binding.

**Figure 5:**
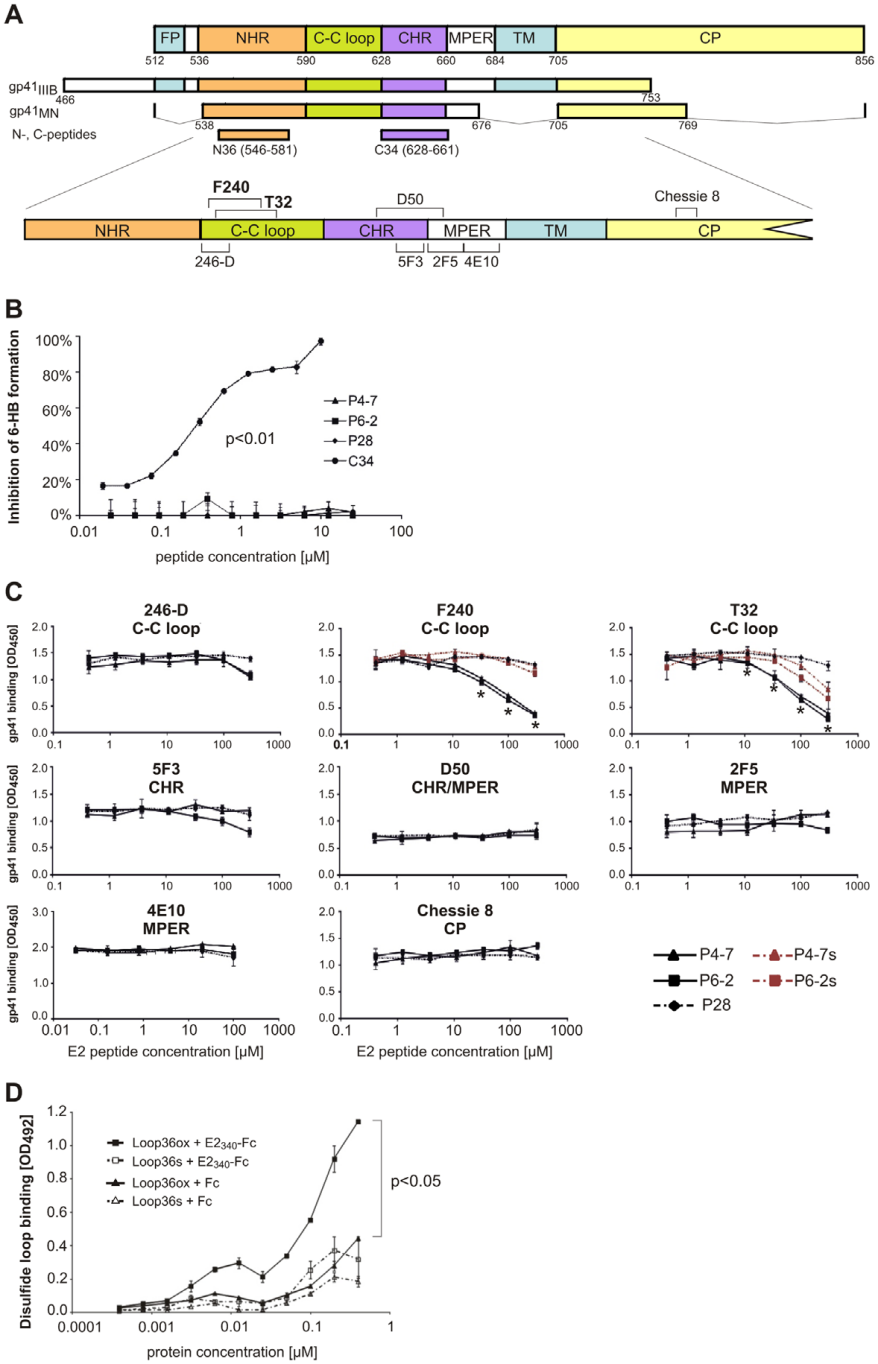
E2-derived peptides bind to the disulfide loop of gp41. (A) Illustration of gp41 variants (strains IIIB, MN) and peptides (N36, C34) used in ELISA as well as the binding epitopes of different monoclonal gp41 antibodies. Gp41 consists of the N-terminal fusion peptide (FP), the N- and C-heptad repeats (NHR, CHR) that are connected by the disulfide loop region (C-C loop), the membrane proximal external region (MPER), the α-helical transmembrane-spanning domain (TM), and the cytoplasmic tail (CP). (B) Inhibitory activity on 6-Helix-Bundle (6-HB) formation of peptides P4-7, P6-2, and P28, as well as C34 as control was measured by ELISA using the NC-1 monoclonal antibody that detects the 6-HB formation between C34-biotin and N36 peptides. Each sample was tested in triplicate. This experiment was repeated twice and similar results were obtained. The statistical significance (*p*<0.01) was achieved for all measure points (C34 vs. P28). (C) Competitive binding of E2 peptides and different gp41 targeting mAbs to immobilized recombinant gp41_MN_. Simultaneously to mAb incubation E2 peptides were added with increasing amounts. The graphs show average values of three independent experiments each performed in duplicate. *: *p*<0.05 (P4-7 or P6-2 vs. P28) (D) Binding of recombinant E2_340_-Fc protein and Fc protein as control to cyclic (Loop36ox) and linear (Loop36s) peptides presenting the HIV-1 gp41 disulfide loop (residues 588–623). The graphs show average values of three independent experiments each performed in duplicate. The statistical significance (*p*<0.05) was achieved with more than 0.003 µM concentrations of each protein (Loop36ox+E2_340_-Fc vs. Loop36ox+Fc).

In order to prove the assumption that GBV-C E2 interacts with HIV Env, subsequent binding assays were performed with plate immobilized subunits of HIV-1 envelope proteins (gp120 and gp41). To improve the significance of this experiment, here we used the whole E2-Fc fusion protein (E2_340_-Fc) as a ligand. The Fc domain alone served as a negative control. Ninety-six-well plates were either coated with recombinant full-length gp120 (gp120_IIIB_ [Immuno Diagnostics, 1001-10], and gp120_MN_ [Immune Technology, IT-001-002MNp]), expressed in eukaryotic cells, or with gp41, expressed in *E. coli*, consisting of the complete (gp41_IIIB_ [Abcam, ab68129]) or truncated gp41 ectodomain (gp41_MN_ [NIH, 12027]). In gp41_MN_, the hydrophobic fusion peptide (FP), the transmembrane region (TM), and most of the C-terminal part of the cytoplasmic tail (CP) were omitted (illustrated in Figure 5A). Whereas no binding of the recombinant E2_340_-Fc fusion protein to gp120 was observed, E2_340_-Fc protein was shown to interact with both gp41 variants in a dose-dependent manner (Figure 4B).

To test whether GBV-C E2 binds HIV gp41 with the N-terminal domain, competition assays were performed with the N-terminal E2 peptides P4-7 and P6-2. The results of this experiment revealed that both HIV-1-inhibitory E2 peptides abrogated the protein-protein interaction between HIV-1 gp41 and GBV-C E2 in a dose-dependent manner, whereas increasing amounts of the negative control peptides P9 and P28 had no effect (Figure 4C, left panel). The E2 peptide P9 (residue 81 to 100 of GBV-C E2, see Table S1) was chosen as an additional control [33], due to its more comparable content of cysteine and hydrophobic residues.

Noteworthy, P4-7 and P6-2 contain either two or three cysteines, respectively. To elucidate the characteristics of the E2-gp41 interaction, next we tested the competition ability of peptide variants P4-7s and P6-2s in which the cysteine residues were replaced with serine. The results revealed that the serine variants P4-7s and P6-2s had lost their ability to abrogate the E2-gp41 binding, suggesting that the cysteine residues within the HIV-inhibitory E2 peptides play a crucial role for gp41 recognition (Figure 4C, right panel).

In combination with these findings, we are able to show that the N-terminal region of GBV-C E2 (∼residue 37 to 64), that is represented by the HIV-1-inhibitory peptides P4-7 and P6-2, interacts with HIV-1 gp41 and that the cysteine residues within this region appear to contribute substantially to the gp41 binding.

### GBV-C E2 Peptides Target the gp41 Disulfide Loop

We next sought to localize the E2 peptide binding site within gp41. The transmembrane protein gp41 consists of several functional regions (Figure 5A). The FP, TM and CP regions of gp41 do not appear to be involved in the interaction with GBV-C E2 or E2 peptides, since the absence of these regions (gp41_MN_) did not abrogate or decrease the E2 binding to gp41 (Figure 4B). To test whether the E2 peptides interact with either the NHR or CHR regions of gp41, which would lead to a blockade of 6-HB formation, a competitive ELISA was performed. In this assay, peptides N36 and biotinylated C34, which present the NHR and the CHR region of gp41, respectively, interact with each other, forming stable 6-HB structures *in vitro* [57], [58]. The 6-HB-specific mAb NC-1 was used as a capture antibody [45] and the GBV-C E2 peptides were tested for competition with N36 and C34, respectively. Neither P4-7 nor P6-2 was able to interfere with the formation of 6-HB, suggesting that the binding site of the E2 peptides is neither located in the NHR (N36) nor in the CHR (C34) regions of gp41 (Figure 5B).

To further dissect the E2 binding site within gp41, we tested whether the E2 peptides compete for gp41 binding with different monoclonal anti-gp41 antibodies with known specificities, including mAbs 246-D, F240, T32, D50, 5F3, 2F5, 4E10, and Chessie 8. Of the eight antibodies, only the gp41 interaction of F240 and T32 was inhibited in a dose-dependent manner by P4-7 and P6-2, but not by the negative control P28 (Figure 5C). No unspecific binding between the E2 peptides (P4-7 and P6-2) and the antibodies F240 or T32 was observed that could have account for the same competition results (data not shown). Therefore, we conclude that P4-7 and P6-2 compete with F240 and T32 considerably for the same binding region. F240 and T32 represent two of three tested antibodies that recognize the immunodominant disulfide loop region of gp41. Their epitopes overlap partially with each other, implying that the E2 peptides bind approximately between residues 596 and 612 of gp160. The epitopes of all tested anti-gp41-mAbs are shown in Figure 5A and Table S2. Again, the serine variants of P4-7 and P6-2 lost their ability to compete for the binding target with the disulfide loop antibody F240 or were at least less efficient (T32). Finally, we performed a direct binding assay with biotin-tagged 36mer gp41 disulfide loop peptides (residues from 588 to 623 according to HIV gp160_HxB2_) and GBV-C E2_340_-Fc. We immobilized via streptavidin an oxidized (closed loop) or a linear variant of the disulfide loop, in which the cysteine residues were replaced with serine (Table S1) onto plates and the binding of graded concentrations of GBV-C E2_340_-Fc was monitored with HRP-anti-human antibodies. Indeed, a distinct binding of recombinant E2_340_-Fc protein to the gp41 disulfide loop peptides was evident in a dose dependent manner, whereas for the Fc control only a low background binding was observed. The binding of E2_340_-Fc was clearly diminished when the linear serine variant of the gp41 disulfide loop was tested (Figure 5D).

### HIV-1 Inhibitory GBV-C E2 Peptides Bear Local Similarities to the gp120 N-terminus that is Part of the gp120-gp41 Interface

**Figure 6:**
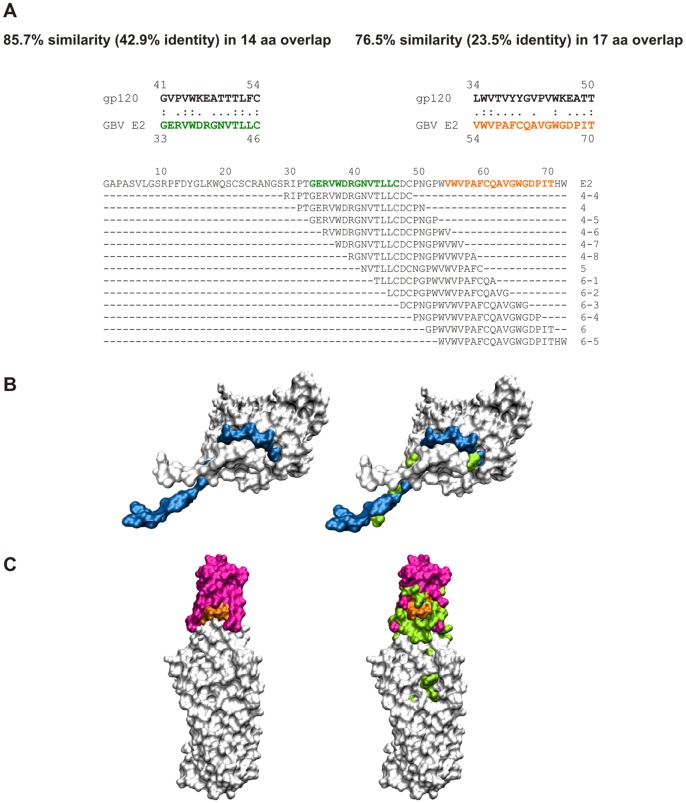
Local similarities between the N-termini of gp120 and E2. (A) Similarities of the two local sequences in the N-termini of HIV-1 gp120 and GBV-C E2. The detected E2 stretches (residues 33–46 and 54–70) are shown in the context of the entire E2 N-terminus and are color coded in green and orange, respectively. Illustrated are the sequences of the peptides that proved to be potent in HIV-1 entry inhibition [33]. These peptides almost exclusively cover the E2 sequence stretch that exhibits similarities to the gp120 N-terminus. Molecules are shown in space-filled presentation and the functionally important regions are colored. (B) Structure of monomeric gp120. The N-terminal region of gp120 that exhibits local similarity with the active E2-derived peptides is shown in blue. Residues that were deduced from mutational analyses to be relevant for the gp120-gp41 interaction (reviewed in [36]) are additionally shown in green. (C) Structure of trimeric gp41. The disulfide-bonded loops that are recognized by the T32 antibody (residues 596–612) are shown in red. Residues 592–596 additionally present in the F240 epitope are shown in orange. Residues that were deduced from mutational analyses to be relevant for the gp120-gp41 interaction (reviewed in [36]) are additionally shown in green. The coordinates for structure presentations were taken from PDB entries 3JWD and 2EZO for gp120 and gp41, respectively.

Specific intra- and intermolecular interactions within HIV-1 Env are essential for the formation of a native and functional HIV-1 envelope trimer, as well as for concerted conformational changes during the entry process. Therefore, peptides or small molecules that mimic Env regions involved in these interactions are potential inhibitors of the formation of properly folded tertiary and quaternary Env structures. Examples of this strategy are potent peptide fusion inhibitors, which resemble the gp41 NHR or CHR sequences, including enfuvirtide (T20) [59], SJ-2176 [60], or N36 [61]. Hence, we performed a similarity search between the GBV-C E2 ectodomain and HIV-1 gp160 and identified a local similarity of two sequence stretches of the GBV-C E2 N-terminus to the HIV-1 gp120 N-terminus (Figure 6A). The sequence stretches 33–46 and 54–70 of E2 exhibit sequence similarities of 85.7% and 76.5% to the N-terminus of gp120. This region of similarity also agrees very well with the sequence region spanned by E2-derived peptides (residues 29–72) that proved to be potent in HIV-entry inhibition [33].

We have also put this sequence similarity in the structural context of gp120 and gp41. High-resolution structural information on gp120-gp41 interaction remains elusive (reviewed in [36]). However, several mutagenesis studies and a recent crystal structure of gp120 suggest that the N- and C-termini of gp120 interact predominantly with the disulfide loop region of gp41 [62], [63]. Interestingly, the N-terminal sequence stretch of gp120 that has been proposed to be involved in the gp120-gp41 interaction is located within the gp120 region that resembles the HIV-1-inhibitory N-terminal GBV-C E2 peptides (Figure 6B). Furthermore, residues within the gp41 trimer, which have been proposed to be involved in the gp120 interface, are located near or within the epitopes of the two antibodies T32 and F240 whose interaction with gp41 was disrupted by the E2 peptides P4-7 and P6-2 (Figure 6C).

**Figure 7:**
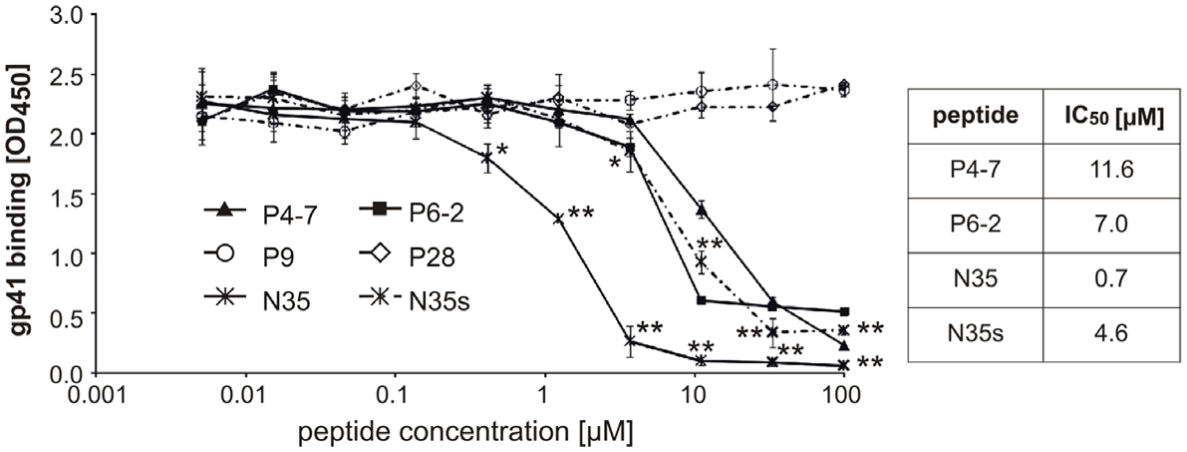
The N-termini of GBV-C E2 and HIV-1 gp120 share the same binding region within the gp41. Competitive binding of E2 and gp120 peptides (N35, N35s) with recombinant E2_340-_Fc protein to immobilized recombinant gp41_MN_. Simultaneously to E2_340_-Fc incubation peptides of E2 or gp120 were added with increasing amounts. The graphs show average values of three independent experiments each performed in duplicate. *: *p*<0.05; **: *p*<0.01 (N35 or N35s vs. P28; P4-7 or P6-2 vs. P28 [data not shown]).

To prove the assumption that a similarity between the GBV-C E2 and HIV gp120 N-termini enables E2 or E2 peptides to bind to gp41 in a gp120 manner, a corresponding N-terminal gp120 peptide should be able to disturb the E2-gp41 interaction. Therefore, we assessed in E2-gp41 competition assays the effect of a gp120 peptide (N35, gp120 residue 31 to 65) that includes the respective E2-similarity region (Figure 7). We observed that N35 efficiently competed with E2 for gp41 binding, implying that E2 and gp120 share the same gp41 interaction site. Not surprisingly, the gp41 affinity was more pronounced for N35 (IC_50_∶0.7 µM) than for the E2 peptides (IC_50_s: 7.0 µM for P6-2 and 11.7 µM for P4-7). The C54S variant of this peptide (N35s) still abrogated the E2-gp41 interaction in a dose dependent manner, but less efficiently than the wild-type N35 peptide (IC_50_∶4.6 µM). Together, these results strongly support the hypothesis that a mimicry phenomenon between the N-termini of the non-related viral glycoproteins E2_GBV-C_ and gp120_HIV,_ enables the E2 N-terminus to approach the gp120 binding site on gp41 that is believed to be the disulfide loop region.

**Figure 8:**
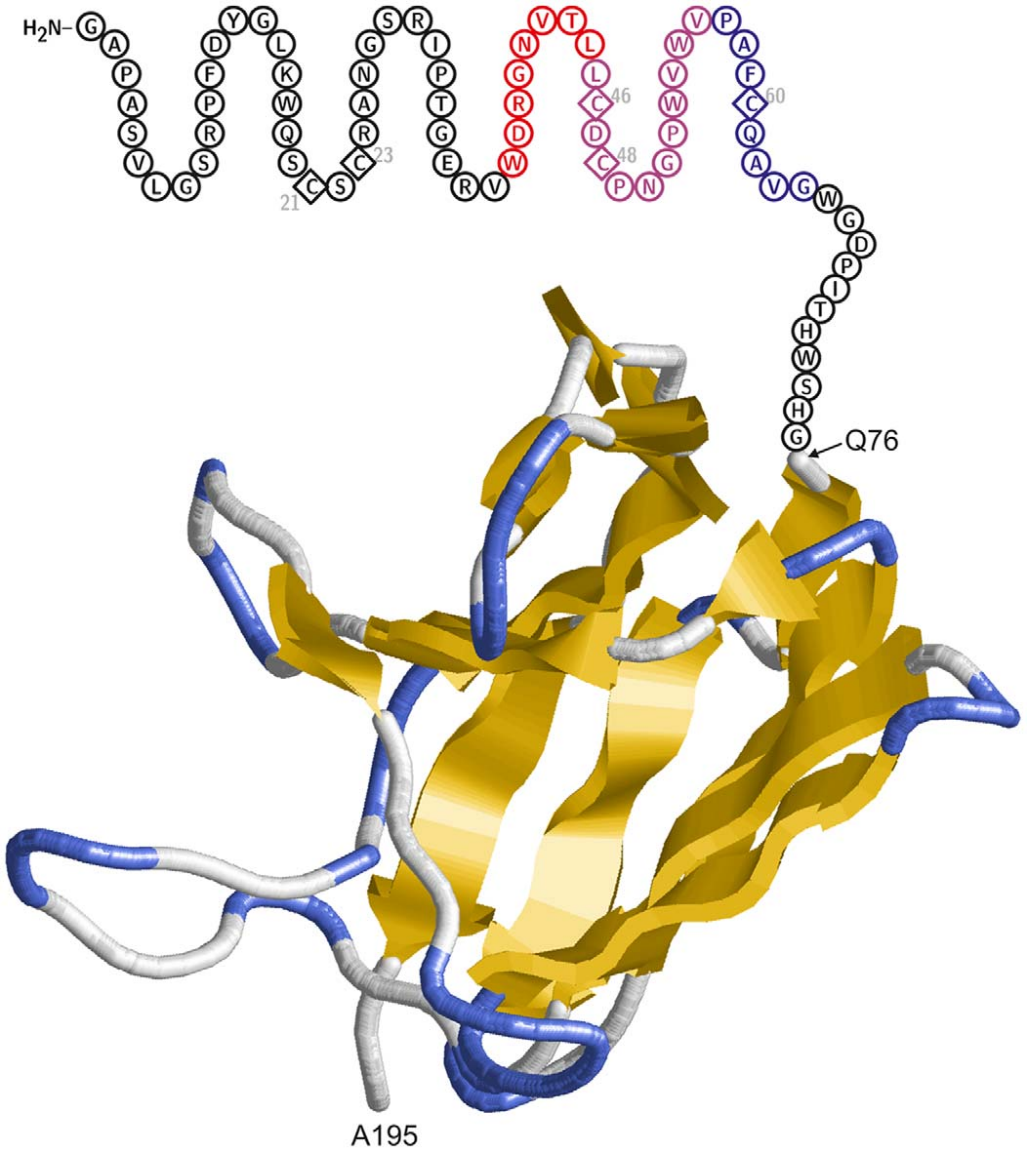
Model of the N-terminal region (residues 1–195) of the E2-protein. The globular Ig-fold domain (residues 76–195), for which a structural model could be generated, is shown in backbone presentation and colored according to the secondary structure type. The N-terminus (residues 1–75), which is not predicted to adopt a globular three-dimensional structure, is schematically depicted as circles indicating the identity of the respective amino acids. Cysteines, which may form disulfide bonds, are shown as diamonds and their sequence position is indicated. Residues belonging to the P4-7 and P6-2 peptides investigated in the present study are highlighted in red and blue, respectively. Overlapping residues present in both peptides are shown in magenta.

Since no structural information on the GBV-C E2 protein is currently available, we performed structure predictions and globularity analyses of the GBV-C E2 protein in order to get an idea whether the N-terminus of E2 is accessible to the interaction with HIV-1 Env involved in membrane fusion. Our analysis consistently suggests the presence of a folded region spanning residues 76 to 195 of GBV-C E2. The remaining extracellular part, in particular the N-terminal region including amino acids 1 to 75 that comprise the HIV-1-inhibitory domain, does not contain significant amounts of regular secondary structure and is therefore predicted to form no globular conformation (Figure 8). Consequently, the E2 N-terminus is likely to be rather flexible which could plausibly account for its interaction with gp41 during HIV-1 entry.

## Discussion

In this study, we have explored the molecular mechanism underlying the previously reported interference with HIV-1 entry by two overlapping peptides derived from the N-terminal part of the GBV-C envelope protein E2. We are able to demonstrate that the E2 peptides interfere with late stages of HIV-1 entry. This notion is evidenced by the observations that neither the cell surface expression of HIV-1 receptors, nor the binding of gp120 to CD4 and coreceptors, respectively, appears to be affected. Instead, HIV-inhibitory E2 peptides showed full activity on HIV entry at a post-binding stage. In agreement with these findings, binding experiments revealed an interaction between E2 or E2 peptides and the HIV-1 transmembrane protein gp41 responsible for late membrane fusion activity.

In an earlier study, we demonstrated that the HIV-1 inhibitory region ranges from residue 29 to 72 of the GBV-C E2 N-terminus [33]. In this study we show that the E2 peptides P4-7 and P6-2 (representing E2 residues 37 to 64) compete with E2 protein for gp41 binding. Therefore, we assume that the HIV-inhibitory region or at least the inner domain ranging from residue 37 to 64 reflects a gp41-binding site of GBV-C E2. Interestingly, the sequence stretch covered by the HIV-inhibitory peptides includes the strongest conserved part of the nonglobular N-terminus of related *flaviviridae* E2 proteins (Figure S1). In particular, several cysteines (C46, C48, C60) and tryptophans (W55, W65) are highly conserved among GBV-C and GBV-A isolates, suggesting that the respective E2 proteins might also exhibit a similar anti-lentiviral activity. The alignment shown in Figure S1 also reveals that the sequence conservation is less pronounced for the more distantly related E2 proteins of GBV-B and GBV-D. The N-termini of the E2 proteins from GBV-C and HCV show no detectable sequence homology at all rendering functional similarity rather unlikely.

The results of several experiments aimed at dissecting the E2-binding domain within gp41 suggest that E2 interacts with the gp41 disulfide loop region. The gp41 disulfide loop region structurally connects the NHR and CHR domains and contains two conserved cysteines [64]. Noteworthy, the N-terminal E2 region contains three cysteine residues (Cys46, Cys48 and Cys60) as well. Previously we could show that variants of the E2 peptides (P4-7s and P6-2s), in which cysteines were replaced with serine residues, lost their HIV-inhibitory capacity [33]. In agreement with these observations, in this study, P4-7s and P6-2s lost their ability to compete with E2 for gp41binding. In addition, the cysteine residues within the disulfide loop peptide appeared to be crucial for the interaction with recombinant E2 protein. This implies that the cysteine residues within the HIV-inhibitory E2 peptides may interfere with the oxidation state of the respective cysteines in the gp41 disulfide loop region. A variety of evidence suggests that a number of viral envelope glycoproteins depend on a dynamic thiol/disulfide balance to mediate virus-cell fusion (reviewed in [65]). For HIV-1 it has been shown that after CD4 binding a cell surface-associated reductase activity leads to cleavage of disulfide bonds at least within gp120 and that this event is obligatory for triggering membrane fusion [66]. However, the insights into the mechanistic role of the disulfide loop cysteines for the fusion reaction are still limited and need further evaluation. Future studies will show, whether reducing agents would change the interference effects of GBV-C E2-derived peptides.

Based on cryo-EM structural information, the gp41 transmembrane protein is expected to be at least partially buried in the trimeric gp41-gp120 structure [67]. Thus, the transient states of gp41 appears to be valid HIV-1 inhibitor targets, as evidenced by a number of known HIV-1 fusion inhibitors, including gp41-targeted peptides and low-molecular-weight inhibitors. These inhibitors typically bind to the NHR or CHR regions during the prehairpin stage in order to prevent the formation of the 6-HB. However, Münch et al. [42] isolated the natural HIV-1 entry inhibitor VIRIP from human hemofiltrates, targeting the gp41 fusion peptide, and broad neutralizing antibodies, like 2F5, 4E10, and Z13e1, bind the MPER, an epitope that is also transiently accessible at a late stage of HIV-1 entry. Our results show that the interaction of a peptide with the gp41 disulfide loop region can block HIV-1 fusion, thus introducing the gp41 disulfide loop as a new and promising target for HIV-1 entry inhibition. The relevance of disulfide loop-targeting HIV-1 inhibitors is further supported by two reports that describe low-molecular-weight HIV-1 inhibitors causing resistance-conferring mutations within that disulfide loop region [68], [69].

The gp41 disulfide loop is an immunodominant region termed cluster I [70], suggesting that this region is permanently or at least transiently accessible [71]. However, unlike the E2 peptides studied here, antibodies directed against cluster I appear to have no relevant HIV-1 neutralizing capacity. The difference of action between the cluster I antibodies and E2 peptides is an interesting question that needs further investigation. One possible explanation may be the fact that steric forces prevent globular antibodies to gain access to the gp41 disulfide loops at the right time, whereas peptides may have sufficient flexibility for this purpose. However, it is tempting to speculate whether *in vivo* the intact E2 ectodomain is able to reach the gp41 disulfide loop in a peptide-like manner? While no structural information on E2 is available, our computer-based structural analysis revealed that the N-terminal region of E2 (residues 1 to 75) appears to be largely unstructured, suggesting that this protein domain may be sufficiently flexible to interact with the disulfide loop of gp41 during HIV-1 entry. In this context, experiments addressing the amount of circulating E2 (virus- versus possibly exosome-associated or processed E2) in plasma of GBV-C coinfected HIV-positive individuals appear appropriate.

The surface glycoprotein gp120 and the transmembrane gp41 subunits are noncovalently associated on the viral surface. However, little is known at the atomic level about the critical gp120-gp41 interface. Several mutagenesis studies suggest that the N- and C-termini of gp120 interact with the disulfide loop of gp41 and flanking parts of the NHR and CHR regions [62], [63], [72]–[74]. However, our knowledge about the dynamic gp120-gp41 interplay during the different phases of HIV-1 entry is sparse. We do not know which parameters allow gp120 to stabilize gp41 in a metastable conformation in the unliganded trimer and after CD4 engagement but support gp41 rearrangements after coreceptor binding. A recent crystal structure of an HIV-1 gp120 core with intact gp120 N- and C-termini revealed three topologically separate and structurally plastic layers [75]. The conformational mobility of these layers together appears to be relevant for buffering movements of gp120 from gp41. It should be noted that the extended gp120 N-terminus appeared particularly flexible, such that interactions with gp41 in the viral spike could easily induce termini movements [75]. This plasticity of the termini might also offer an explanation for the fact that the interactions of the gp120 N-terminus with gp41 can be mimicked by flexible peptides. Computational and experimental data from this study indicate that N-terminal GBV-C E2 peptides mimic the HIV-1 gp120 N-terminus and are therefore able to compete with gp120 for binding to the disulfide loop region of gp41. Competition experiments confirmed that E2 peptides (P4-7 and P6-2) and a corresponding N-terminal gp120 peptide ranging from residue 31 to 65 of gp120 share the same binding region in gp41. Therefore, we conclude that the respective E2 peptides may displace gp120 and disturb the interface between gp120 and gp41 after CD4 or coreceptor binding. This phenomenon may result in impeding the concerted interplay between stabilization (native, unbound state) and destabilization (receptor-bound and following states) of the gp120-gp41 complex, which is critical for HIV-1 entry to take its proper course. In particular, binding of E2 or E2-derived peptides to the gp41 disulfide loop may influence the proper disulfide formation of gp41, the flexibility of gp41 that is needed to promote the 6-HB formation, or the membrane accessibility of gp41 that is essential for lipid mixing. However, the elucidation of the precise mode of action of the disulfide loop targeting peptides will be an interesting subject for future evaluations. In this context, it would be interesting to consider chimeric peptides that incorporate sequences of GBV-C E2 and the HIV-1 N-terminus of gp120 as a new approach to enhance the antiretroviral activity of disulfide loop-targeted peptide inhibitors.

Previously, Herrera et al. [76] reported that peptides derived from the N-terminus of GBV-C E2 (ranging from residues 31 to 78), as well as several more downstream regions of E2, interact with the HIV-1 fusion peptide, resulting in suppression of HIV-1 replication. Our data do not confirm the association of the GBV-C E2 N-terminus with the HIV-1 fusion peptide during HIV-1 entry. However, it is very plausible that other E2 regions interact with the N-terminal gp41 fusion peptide, in addition to the interaction of the GBV-C E2 N-terminus with the gp41 disulfide loop proposed in this study. Most recently, Xiang et al. [35] reported that the GBV-C E2 protein or a peptide representing the E2 residues 276 to 292 interfered with HIV-1 entry, when expressed in Jurkat cells. However, when added to cells the respective peptide did not inhibit HIV-1 entry unless it was fused to Tat for cellular uptake. Taken together, these observations support the relevance of GBV-C E2 for HIV-1 entry interference but suggest that additionally to the N-terminus at least the C-terminal part of the E2 ectodomain is involved in HIV-1 entry inhibition. This may also explain the modest effect on CXCR4 cell surface presentation upon expression of full length E2 that we do not see after cell incubation with N-terminal E2 peptides. However, it needs further evaluation to what extent E2-presenting GBV-C particles or GBV-C infected cells via a bystander effect, proposed by Xiang et al. [35], contribute to the E2-mediated HIV-1 entry impairment.

In conclusion, we propose a new strategy of HIV-1 entry inhibition based on sequence resemblance between the N-termini of GBV-C E2 and HIV-1 gp120. Our results demonstrate that peptides targeting the gp41 disulfide loop are able to inhibit HIV-1 fusion, introducing a novel design concept for HIV-1 fusion inhibitors.

## Supporting Information

Figure S1 **Multiple sequence alignment of related E2 proteins.** Multiple sequence alignment of E2 proteins from GBV-C (black), GBV-A (green), GBV-B (red), and GBV-D (blue). The GBV-C isolate used in the present study is shown in the first line and the sequence stretch covered by the active peptides is underlined. The second line shows a distantly related E2 protein from chimpanzee GBV-C to highlight the sequence divergence within the GBV-C isolates. Conserved cysteines and aromatic residues are shown as bold letters.
(TIF)

Table S1 **Sequences of synthetic GBV-C E2 and HIV-1 peptides.** *Numbering follows GBV-C E2 GenBank accession no. AF121950 or HIV-1_HXB2_ gp160 (HIV databases: http://www.hiv.lanl.gov), respectively X: ε-aminohexanoic acid.
(DOC)

Table S2 **Specificities of gp41 targeting antibodies.** *Numbering follows HIV-1_HXB2_ gp160 (HIV databases: http://www.hiv.lanl.gov). Bold letters represent the core of the epitope, and flanking amino acids may contribute to binding efficiency.
(DOC)
