# Correction: HIV-1 Fusion Is Blocked through Binding of GB Virus C E2D Peptides to the HIV-1 gp41 Disulfide Loop

**DOI:** 10.1371/annotation/b46459e1-73ff-4b75-8f24-264cbcf4a5a9

**Published:** 2014-01-13

**Authors:** Kristin Eissmann, Sebastian Mueller, Heinrich Sticht, Susan Jung, Peng Zou, Shibo Jiang, Andrea Gross, Jutta Eichler, Bernhard Fleckenstein, Heide Reil

The word "E2D" is misspelled in the article title. The correct title is: HIV-1 Fusion is Blocked through Binding of GB Virus C E2-derived Peptides to the HIV-1 gp41 Disulfide Loop.

The correct citation is: Eissmann K, Mueller S, Sticht H, Jung S, Zou P, et al. (2013) HIV-1 Fusion is Blocked through Binding of GB Virus C E2-derived Peptides to the HIV-1 gp41 Disulfide Loop. PLoS ONE 8(1): e54452. doi:10.1371/journal.pone.0054452 

