# Supplementary figures and images for: HIV-1 Fusion Is Blocked through Binding of GB Virus C E2D Peptides to the HIV-1 gp41 Disulfide Loop
Source: PLoS One. 2013 Jan 22;8(1):e54452. doi: 10.1371/journal.pone.0054452 (PMC3551756; doi:10.1371/journal.pone.0054452)

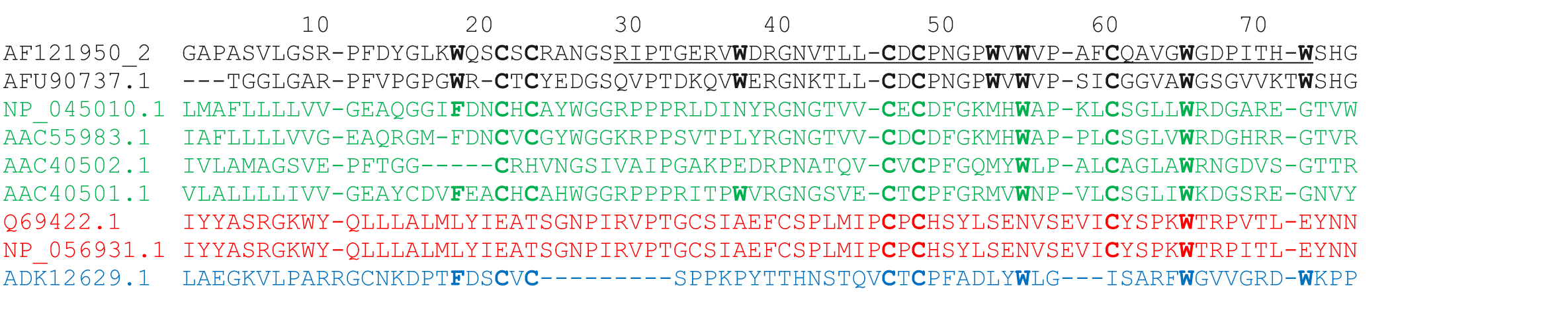

Supplement: Figure S1 — Multiple sequence alignment of related E2 proteins. Multiple sequence alignment of E2 proteins from GBV-C (black), GBV-A (green), GBV-B (red), and GBV-D (blue). The GBV-C isolate used in the present study is shown in the first line and the sequence stretch covered by the active peptides is underlined. The second line shows a distantly related E2 protein from chimpanzee GBV-C to highlight the sequence divergence within the GBV-C isolates. Conserved cysteines and aromatic residues are shown as bold letters. (TIF) [file pone.0054452.s001.tif]
