# Supplementary material for: HIV-1 Fusion Is Blocked through Binding of GB Virus C E2D Peptides to the HIV-1 gp41 Disulfide Loop
Source: PLoS One. 2013 Jan 22;8(1):e54452. doi: 10.1371/journal.pone.0054452 (PMC3551756; doi:10.1371/journal.pone.0054452)
Supplement: Table S1 — Sequences of synthetic GBV-C E2 and HIV-1 peptides. *Numbering follows GBV-C E2 GenBank accession no. AF121950 or HIV-1HXB2 gp160 (HIV databases: http://www.hiv.lanl.gov), respectively X: ε-aminohexanoic acid. (DOC) [file pone.0054452.s002.doc]

Table S1: Sequences of synthetic GBV-C E2 and HIV-1 peptides

| **peptide** | **residue positions**  **GBV-C E2*** | **sequence** |
| --- | --- | --- |
| P4-7 | 37-56 | Ac-WDRGNVTLLCDCPNGPWVWV |
| P6-2 | 45-64 | Ac-LCDCPNGPWVWVPAFCQAVG |
| P9 | 81-100 | Ac-LSCPQYVYGSVSVTCVWGSV |
| P28 | 271-290 | Ac-TEVSEALGGAGLTGGFYEPL |
| P4-7s | 37-56 | Ac-WDRGNVTLLSDSPNGPWVWV |
| P6-2s | 45-64 | Ac-LSDSPNGPWVWVPAFSQAVG |
|  | **HIV-1*** |  |
| N35 | 31-65 | Ac-EKLWVTVYYGVPVWKEATTTLFCASDAKAYDTEV-NH_2_ |
| N35s | 31-65 | Ac-EKLWVTVYYGVPVWKEATTTLFSASDAKAYDTEV-NH_2_ |
| Loop36ox | 588-623 | Biotin-X-KDQQLLGIWGCSGKLICTTAVPWNASWSNKSLEQIW-NH_2_ |
| Loop36s | 588-623 | Biotin-X-KDQQLLGIWGSSGKLISTTAVPWNASWSNKSLEQIW-NH_2_ |

*Numbering follows GBV-C E2 GenBank accession no. AF121950 or HIV-1_HXB2_ gp160 (HIV databases: http://www.hiv.lanl.gov), respectively

X: ε-aminohexanoic acid
