# Supplementary material for: HIV-1 Fusion Is Blocked through Binding of GB Virus C E2D Peptides to the HIV-1 gp41 Disulfide Loop
Source: PLoS One. 2013 Jan 22;8(1):e54452. doi: 10.1371/journal.pone.0054452 (PMC3551756; doi:10.1371/journal.pone.0054452)
Supplement: Table S2 — Specificities of gp41 targeting antibodies. *Numbering follows HIV-1HXB2 gp160 (HIV databases: http://www.hiv.lanl.gov). Bold letters represent the core of the epitope, and flanking amino acids may contribute to binding efficiency. (DOC) [file pone.0054452.s003.doc]

**Table S2: Specificities of gp41 targeting antibodies**

| **antibody** | **epitope** | **sequence gp160^*^** | **references** |
| --- | --- | --- | --- |
| 246-D | disulfide loop | 590-597  [QQ**LLGI**WG] | [1] |
| F240 | disulfide loop | 592-606  [LLGIWGCSGKLICTT] | [2] |
| T32 | disulfide loop | 596-612  [WGCSGKLICTTAVPWNA] | [3] |
| 5F3 | CHR | 650-657  [QNQQEKNE] | Polymun Scientific, Austria |
| D50 | CHR/MPER | 642-665 [IHSLIEESQNQQEKNEQELLELDK] | [3] |
| 2F5 | MPER | 660-670  [LL**ELDKWAS**LW] | [4,5] |
| 4E10 | MPER | 671-680  [NWFDITNWLW] | [6] |
| Chessie 8 | CP | 727-732  [PDRPEG] | [7] |

*Numbering follows HIV-1_HXB2_ gp160 (HIV databases: http://www.hiv.lanl.gov). Bold letters represent the core of the epitope, and flanking amino acids may contribute to binding efficiency
